# A Community Study of Factors Related to Poorly Controlled Asthma among Brazilian Urban Children

**DOI:** 10.1371/journal.pone.0037050

**Published:** 2012-05-31

**Authors:** Silvia de Magalhães Simões, Sergio S. da Cunha, Álvaro A. Cruz, Karen Conceição Dias, Neuza M. Alcântara-Neves, Leila D. Amorim, Laura C. Rodrigues, Maurício L. Barreto

**Affiliations:** 1 Centro de Ciências Biológicas e da Saúde, Universidade Federal de Sergipe, Aracaju, Brazil; 2 SCAALA (Social Change, Asthma and Allergy in Latin America) Study Group; 3 Departamento de Medicina Social, Universidade Federal de Pernambuco, Recife, Brazil; 4 ProAR – Núcleo de Excelência em Asma da Universidade Federal da Bahia (PRONEX CNPq/FAPESB), Salvador, Brazil; 5 Instituto de Saúde Coletiva, Universidade Federal da Bahia, Salvador, Brazil; 6 Instituto de Ciências da Saúde, Universidade Federal da Bahia, Salvador, Brazil; 7 London School of Hygiene and Tropical Medicine, London, United Kingdom; 8 Instituto de Matemática, Universidade Federal da Bahia, Salvador, Brazil; Johns Hopkins University, United States of America

## Abstract

**Background:**

Asthma constitutes a serious public health problem in many regions of the world, including the city of Salvador, State of Bahia – Brazil. The purpose of this study was to analyse the factors associated with poor asthma control.

**Methodology/Principal Findings:**

Two definitions were used for asthma: 1) wheezing in the last 12 months; 2) wheezing in the last 12 months plus other asthma symptoms or asthma diagnosis ever. The definition of poorly controlled asthma was: at least one reported hospitalisation due to asthma and/or high frequency of symptoms, in the last year. Children with poorly controlled asthma (N = 187/374) were compared with wheezing children with controlled asthma regarding age, gender, atopy, parental asthma, rhinitis, eczema, exposure to second hand tobacco smoke, presence of moulds, pets and pests in the house, helminth infections and body mass index. Crude and logistic regression adjusted odds ratios were used as measures of association. There was a higher proportion of poorly controlled asthma among children with eczema (OR = 1.55; 95% CI 1.02; 2.37). The strength of the association was greater among children with eczema and rhinitis (42.6%, 53.4% and 57.7%, respectively, in children who had no rhinitis nor eczema, had only one of those, and had both (p = 0.02 for trend test). The presence of mould in the houses was inversely associated with poorly controlled asthma (OR = 0.54; 95% CI 0.34; 0.87).

**Conclusions/Significance:**

Our results indicate an association between eczema and poor asthma control in this environment, but emphasize the role of various other individual and environmental factors as determinants of poor control.

## Introduction

Asthma is the most common chronic disease among children in many regions including developing countries [1] and often constitutes a serious public health problem because of its morbidity and costs. Studies have shown an increase in the prevalence of asthma in several countries in the last decades [2]. Surveys conducted in various regions of the world demonstrated that most subjects with asthma have symptoms or limitations of their daily activities related to lack of control of their disease [3]. Recently, The Asthma Insights and Reality in Latin America Study (AIRLA) showed that more than 50% of the interviewees had been hospitalized for asthma, attended a hospital emergency service or made an unscheduled medical visit due to asthma within the previous year [4].

Several factors seem to be associated with increased prevalence or severity of asthma. Asthmatic children chronically exposed to aeroallergens (e.g. mites, cockroaches, pet allergens) appear to have more severe asthma [5], [6]. Analysis of the US Third National Health and Nutrition Examination Survey found that children with asthma of greater severity were more frequently exposed to second hand smoke, as compared with those with milder asthma [7]. Sensitisation to moulds is another factor that has been related to severity of asthma in adults [8]–[10]. Exposure to Alternaria allergens was associated to the severity of asthma in children, especially in regions where exposure to this fungus is high [11].

Asthma and rhinitis are characterized by similar pathophysiological mechanisms and are often observed in the same patient [12]. Previous studies have suggested that the co-existence of rhinitis is related to uncontrolled asthma [13]–[15]. Moreover, the presence of both rhinitis and eczema in children with asthma is associated with increased severity of the lower airway disease [13]. Obesity has also been shown to be associated with asthma of greater severity: a recent cross-sectional study of adolescents living in the southern region of Brazil showed that there is a positive direct association between obesity and prevalence of asthma symptoms [16].

In low-income settings helminth infection may be related to asthma. The inter-relationship between helminth infections and asthma is controversial, however. In Brazil reports indicated *Schistosoma mansoni* infection is a factor of protection against atopy [17] and a factor related to attenuated severity of asthma [18]. Nevertheless, in a comparison of subjects with asthma and/or allergic rhinitis according to the presence of *Ascaris lumbricoides* infection we found no association with asthma severity nor skin reactivity to aeroallergens [19].

The aim of the present study was to evaluate the frequency of uncontrolled asthma in a population based sample of children of the City of Salvador, Brazil, and to investigate factors associated with poor control of the disease.

## Methods

### Study subjects

1445 children aged 4–11 years, living in 24 small defined areas in the City of Salvador, Northeast of Brazil were selected and studied in 2005. The children's guardians received a letter with information about the study and children participated if their guardians accepted the consent letter and also provided written consent. The study was approved by the Institutional Review Board (Comitê de Ética em Pesquisa do Instituto de Saúde Coletiva da Universidade Federal da Bahia) and subsequently by the National Commission for Ethics in Research (CONEP, Brazil) in 2004. Specialised medical care was offered to all children classified as having severe asthma by a trained paediatrician in an outpatient referral clinic.

### Study design

This was a cross-sectional study conducted as the baseline survey of a cohort study aimed to assess risk factors for asthma and allergic diseases, described elsewhere [20].

### Methods

#### Questionnaires and definitions

The ISAAC phase II standard questionnaire, translated into Brazilian Portuguese, was applied by trained field workers in 2005. Questions on asthma symptoms were used to define the cases of asthma and its severity. We used two different definitions for current asthma. The first – wheezing in the last 12 months; and the second, a more specific definition, included wheezing in the last 12 months plus at least one of the following: history of asthma ever, ≥4 wheezing episodes in the last 12 months, wheezing with exercise in the last 12 months, and >1 sleep disordered due to wheezing in the last 12 months.

We also used two different criteria for asthma control: 1) based on symptoms, poorly controlled asthma was defined as cases having at least one of the following in the last 12 months: ≥12 wheezing episodes, wheezing and breathlessness resulting in difficulty in speaking, and >1 day of disturbed sleep/week due to asthma; 2) cases were also considered poorly controlled if they had at least one hospitalisation because of asthma in the last year. Children fulfilling the symptoms criteria and/or having the hospitalisation criteria were classified as having poorly controlled asthma.

#### Potential risk factors for poor control of asthma

The following variables were registered in 2005 and subsequently analysed: age, gender, skin prick test and specific IgE for common allergens, parental asthma, rhinitis, flexural skin lesions suggestive of eczema, second hand cigarette smoke at home, presence of moulds in the house upon inspection, presence of pets (cat or dog) and pests (rats or cockroach) in the house, helminth infections (*A. lumbricoides* and *T. trichiura*), body mass index (eutrophic, overweight/obesity, deficit) and mite detected on the dust of the child's bed.

The presence of symptoms suggestive of allergic rhinitis was defined as nasal symptoms (sneezing, runny nose and congestion) accompanied by itchy-watery eyes in the last 12 months not associated to colds. The presence of flexural skin lesions suggestive of eczema was defined by self-report as the presence of itchy rash at any time affecting any of the following places: the folds of the elbows, behind the knees, in front of the ankles, under the buttocks, or around the neck, ears or eyes in the last 12 months.

#### Helminth infections

Two stool samples were collected in 2005 for helminth and protozoan identification. Stools were analysed using a gravitational sedimentation technique [21]. Quantification of helminth eggs was performed using Kato-Katz technique [22] and the concentration was expressed as number of eggs per gram of feces.

#### Atopy

Blood samples were collected for assays of allergen specific IgE to *D. pteronyssinus*, *B. tropicalis*, *B. germanica and P. americana* by Immuno-cap system (Pharmacia, Upsala, Sweden). The cutoff for a positive result was 0.35 KU/IgE/L. Skin prick test (SPT) was carried out in the right forearm of each child using extracts of *Dermatophagoides pteronyssinus*, *Blomia tropicalis*, *Blatella germanica, Periplaneta americana*, a pool of fungi (*Aspergillus amstelodami, A. fumigatus, A. niger, A. terrus, Penicillium brevicompactum, P. expansum, P. notatum, P. roquefotii, Cladosporium fulvum and C. Herbarum*), dog and cat epithelia (ALK-Abello, São Paulo, Brazil). Saline and histamine were used as negative and positive controls and wheal sizes were read after 15 minutes of puncture. SPT was considered positive if the mean of two perpendicular diameters of the wheel at each puncture site of any allergen was ≥3 mm greater than the mean diameter of the individual negative control. A child was considered atopic when he or she had levels of at least one allergen specific IgE>0.35 UK or a positive SPT.

#### Obesity

A child was considered overweight or obese when the body mass index (BMI)≥85^th^ percentile for age and sex, and with deficit when BMI was below percentile 5^th^ [23].

#### Dust mite in bed

Dust samples were collected using a residential vacuum cleaner (Electrolux Professional, 1220 watts, São Paulo, Brazil) containing a nylon 25 um micromesh sock filter [24]. Children mattresses were aspirated for two minutes over a 1 m^2^ area adjacent to the head side. The Der p 1 and Blo t 5 allergens were quantified by capture ELISAs using commercially available kits (Indoor Biotechnologies, Virginia, USA) following the manufacturer's protocols. Allergen concentration was expressed as micrograms per gram of dust (µg/g).

### Statistical analysis

The analysis reported here is restricted to children classified as having a history of wheezing in the previous 12 months in this survey. Descriptive statistics of the variables of interest are presented. Comparison of the characteristics of the sample with complete data to those individuals with missing data is performed using chi-square test, Fisher exact test or t-Student test where appropriate. Prevalences of poorly control asthma were computed and compared based on demographic, biological characteristics and on clinical characteristics. Chi-square trend tests were applied for evaluating the effect of number of symptoms (rhinitis and eczema) on the prevalence of poorly controlled asthma. Logistic regression models are implemented for measuring the strength of the association between study variables and poorly controlled asthma. The estimates for the odds ratios were adjusted for children's age and gender, as well as for parental asthma status. Crude and adjusted odds ratio (and corresponding 95% confidence intervals) are presented. Statistical analysis was performed using SPSS version 15.0 software (SPSS Inc., Chicago, IL, USA).

## Results

From the survey of 1445 children, the guardians of 417 (28.9%) reported that the children had wheezing in the last 12 months; of these, 374 (89.7% of 417) had complete data set and were included in the present analysis. None of wheezing children was receiving controller medications, most had rescue oral bronchodilators for use during exacerbations as their only treatment [25]. Regarding the socioeconomic status, 43.4% of mothers had not completed high school (160/369), the predominant type of constructions was brick houses (354/370, 95.7%) with piped water and sewage system present in 82.7% and 83.6% of them, respectively.

Table 1 compares the 374 children analysed and the 43 excluded from analysis due to missing information. The children analysed presented a slightly higher proportion of males, eutrophic and overweight/obese, helminth infections in 2005, presence of cat or dog, presence of mice or cockroaches and dust mite allergens detected on bed. Those excluded presented a higher proportion of other characteristics. However, the differences were of small magnitude and none of the differences observed was statistically significant (Table 1).

**Table 1:** Characteristics of the subjects within the sample analysed and subjects excluded from analysis.

Study variable		Sample analysed N = 374		Subjects excludedN = 43		p value^1^
		N	%	N	%	
Male gender - n (%)		199	53.2	21	48.8	0.587
Age (years)	percentiles (25, 50 and 75)	5.07/6.01/7.47		5.03/5.88/6.99		0.238^2^
	mean (SD)	6.37 (1.68)		6.05 (1.43)		
Body mass categories (n)	eutrophic	284	75.9	29	67.4	
	Overweight/obese	49	13.1	5	11.6	0.163^3^
	underweight	41	11.0	9	20.9	
History of flexural skin lesions (eczema)		147	39.3	19	44.2	0.536
History of symptoms of rhinitis		157	42.0	20	43.5	0.569
Mould at inspection of the house		270	72.2	33	78.6	0.378
Current smoker at home		102	27.3	16	38.1	0.114
Atopy		226	60.4	15/22*	65.2	0.648
Parental Asthma		75	20.1	11/40*	27.5	0.270
Helminth infections in 2005		114	30.5	9/35*	25.7	0.556
Presence of cat or dog		112	30.0	11	25.6	0.552
Presence of mice or cockroaches		335	89.6	36	83.7	0.246
Dust mite allergens detected on bed		222	59.4	14/30*	46.7	0.175

Note:

1 p value based on *chi* square, and Fisher if the n in a cell <5;

2 *t* test for age;

3 joint p value.

* The second number refers to the total number of subjects with data for the corresponding variable.

Among the 374 wheezing children with a complete data set, 187 (50.0%) children were classified as having poorly controlled asthma. Among the poorly controlled, 107 (57.2%) had symptoms of greater severity but no hospitalisation, 30 (16.0%) had hospitalisations but no symptoms of greater severity, and 50 (26.7%) had both. Table 2 presents the association (crude and adjusted odds ratio) between the study exposures and presence of poorly controlled asthma among the 374 asthmatic children. There was a significantly higher proportion of poor control among those children with history of eczema (OR = 1.55; 95% CI 1.02; 2.37). The presence of mould in the houses was associated with a lower proportion of cases with poor control (OR = 0.54; 95% CI 0.34; 0.87).

**Table 2:** Crude and adjusted odds ratios for the relation between study variables and poor control of asthma.

Study variables		Asthma poorly controlled	Crude OR and 95%CI	Adjusted OR and 95%CI
**Gender**	Female	79/175(45.1)	1	1
	Male	108/199(54.3)	1.44 (0.96;2.17)	1.44 (0.96;2.18)
**Age groups**	3–4–5 y	98/186 (52.7)	1	1
	6–7–8 y	70/153 (45.8)	0.76 (0.50;1.16)	0.74 (0.48;1.14)
	9–10–11 y	19/35 (54.3)	1.07 (0.52;2.20)	1.05 (0.51;2.18)
**Body mass (n/N)**	Eutrophy	146/284 (51.4)	1	1
	Overweight/Obese	23/49 (46.9)	0.84 (0.46;1.53)	0.88 (0.48;1.62)
	Deficit	18/41 (43.9)	0.74 (0.38;1.43)	0.73 (0.38;1.43)
**Flexural skin lesions**	No	**104/227 (45.8)**	1	1
	Yes	**83/147 (56.5)**	**1.53 (1.01;2.33)**	**1.55 (1.02;2.37)**
**Rhinitis**	No	101/217 (46.5)	1	1
	Yes	86/157 (54.8)	1.39 (0.92;2.10)	1.40 (0.92;2.13)
**Flexural skin lesions**	None	63/148 (42.6)	1	1
**and Rhinitis**	Only one	79/148 (53.4)	1.54 (0.98;2.44)	1.58 (1.00;2.51)
	Both	45/78 (57.7)	**1.84 (1.05;3.20)**	**1.85 (1.05;3.24)**
**Atopy**	No	67/148 (45.3)	1	1
	Yes	120/226 (53.1)	1.37 (0.90;2.07)	1.35 (0.88;2.06)
***Trichuris*** ** infection**	No	150/300 (50.0)	1	1
	Yes	37/74 (50.0)	1.00(0.60; 1.66)	1.02(0.61; 1.70)
***Ascaris*** ** infection**	No	149/297 (50.2)	1	1
	Yes	38/77 (49.4)	0.97(0.59; 1.60)	0.98(0.59;1.62)
**Parental asthma**	No	148/299 (49.5)	1	1
	Yes	39/75 (52.0)	1.10 (0.67;1.83)	1.12 (0.67;1.87)
**Mould (home inspection)**	No	**63/104 (60.6)**	1	1
	Yes	**124/270 (45.9)**	**0.55 (0.35;0.88)**	**0.54 (0.34;0.87)**
**Exposure to second hand smoke at home**	No	129/272 (47.4)	1	1
	Yes	58/102 (56.9)	1.46 (0.92;2.31)	1.52 (0.95;2.42)
**Presence of cat at home**	No	167/332 (50.3)	1	1
	Yes	20/42 (47.6)	0.90 (0.47;1.71)	0.89 (0.47;1.71)
**Presence of dog at home**	No	142/288 (49.3)	1	1
	Yes	45/86 (52.3)	1.13 (0.70;1.83)	1.13 (0.69;1.86)
**Occasional presence of mice in the household**	No	76/145 (52.4)	1	1
	Yes	111/229 (48.5)	0.85 (0.56;1.30)	0.82 (0.54;1.25)
**Occasional presence of cockroach in the household**	No	29/65 (44.6)	1	1
	Yes	158/309 (51.1)	1.30 (0.76;2.22)	1.25 (0.73;2.16)
**Dust mite allergens detected on bed**	No	76/152 (50.0)	1	1
	Yes	111/222 (50.0)	1.00 (0.66;1.50)	1.00 (0.66;1.52)

Bolding indicates statistically significant results.

We found a significant trend to a higher frequency of poorly controlled asthma in children with eczema and rhinitis combined. Prevalences were 42.6%, 53.4% and 57.7%, respectively, when children had no rhinitis nor eczema, had only one of those, and had both (p = 0.02 for trend test). The association between poorly controlled asthma and the combination of eczema and rhinitis was maintained even after control for other factors through logistic regression (Table 2).

The proportion of atopy was similar among asthmatics with rhinitis and no rhinitis (62.4% *vs* 59.0%, p = 0.503) and eczema or no eczema (61.4% *vs* 59.9%, p = 0.800).

The analysis was subsequently restricted to those with current asthma defined by history of wheezing in the last 12 months and at least one of the other indicators for greater specificity (n = 291). In this subset of 291 children, 181 (62.2%) were classified as having poorly controlled asthma. The analysis showed results very similar to the total 374 children with a history of wheezing and thus were not presented in a separate table.

## Discussion

In this study, we took opportunity of a survey to conduct an analysis in the subsample restricted to those with asthma symptoms and explored the relationship between several potential risk factors to poorly controlled asthma. The main findings of this study were: (i) 50.0% of children with a history of wheezing in the last 12 months had symptoms of greater severity and/or at least one hospitalisation of asthma, and were considered poorly controlled; (ii) the presence of eczema alone or in conjunction with rhinitis was associated with asthma of poor control; (iii) the presence of mould in the house was inversely associated with poorly controlled asthma.

Eczema has been previously associated with asthma severity, including one study in Brazil [13]. In another report, bronchial hyperresponsiveness and eosinophilic airway inflammation were significantly more common in patients with moderate to severe eczema, especially those with positive SPT and high levels of IgE, than in control subjects without eczema [26]. The authors argue that eosinophils activated in eczema might contribute to airway inflammation in these patients. In our study, however surprisingly we found no association between atopy and flexural skin lesions, but the association between flexural skin lesions and severe asthma remained even after controlling for atopy. These results reinforce the concept of multicausality of eczema, and stresses the possibility that asthma and eczema may share other mechanisms beyond atopy.

The association between indoor mould exposure and asthma has been described in several studies, but few studies analysed the association between mould and asthma severity or control. Indeed, there is some evidence that sensitisation and exposure to certain moulds is associated with asthma exacerbations, which is related to control. In a study in Australia, asthmatic children with sensitisation to *Alternaria sp* had more symptoms and used more often bronchodilator than those without sensitisation [11]. If mould exposure triggers symptoms of asthma, it should be associated with severity among subjects who are sensitized. But there are no consistent reports on this aspect in the literature: a case-control study based on 763 children in the UK, which used the criteria of the ISAAC questionnaire to define asthma severity, did not find an association between mould exposure and asthma severity [27]. In our study, the presence of indoor mould exposure was assessed by inspection by the interviewer and was significantly inversely associated with poor control of asthma. We speculate that this finding could be due to reverse causation: the guardians of asthmatic children with poorly controlled asthma would take more actions to remove mould from the homes or move away from homes with mould, assuming guardians were concerned with mould. We questioned about the presence of mould at home in a second survey of the same population conducted in 2007 and we observed a significant reduction of mould in homes of wheezing children. However, further studies are needed to prove that the removal of mould reduces the severity of asthma in this population. Finally, mould was found during home inspection in 72% of the 374 children, but only 5 children (1.33%) were found sensitised to the fungi allergens used in our SPTs.

In order to evaluate the relationship between rhinitis and eczema with the severity of asthma, we analyzed the risk of asthma of poor control related to either one of them or both combined, in comparison to that of subjects with neither rhinitis nor eczema and found a significantly higher proportion for poorly controlled asthma among children with one of both (rhinitis and eczema) and the highest proportion among children who had both. Applying the ISAAC questionnaire, Solé et al reported the association of rhinitis or eczema and the severity of asthma [13]. In our study, the proportion of atopy was not higher among asthmatics with rhinitis and eczema, as compared with those without these comorbities. Therefore atopy could not explain the association described. Further research is needed to determine if the effect of eczema on asthma control is independent of atopy.

For the correct interpretation of our results, one should consider that we did not use data from medical records nor any complementary test at the moment of the survey, such as spirometry. Instead, we analysed self-reported data and thus they are susceptible to misclassification. Given that there have been doubts on the accuracy of the common definition of asthma and its control based on self reported symptoms [28], [29], we have tried to minimise such a bias by using a second and more accurate definition for asthma and two criteria for poor control. It is worth noting that different definitions for asthma control have been used in the literature [13], [27], and along with different interpretations of the questions due to language variation, what makes comparison of results more difficult. This is a population-based study. The sample size for children having asthma (374 out of 1445 surveyed) offer limited power for certain inferences indeed. However, we have a community survey among children with abundant information on potential risk factors, protection factors and outcomes, which are crucial for a more accurate assessment of confounders and consider our methods to be the major strength of this report. In exploring this complex issue, only a few studies [27] have managed to perform a comprehensive evaluation of a population-based sample to include multiple potential risk and protection factors.

In conclusion, we identified that eczema increases significantly the chance of poorly controlled asthma among asthmatic children identified in a population survey in a low-income setting. Subjects presenting eczema and concomitant symptoms of rhinitis have a further increase in the risk of poor control of their asthma as compared with individuals having asthma but none of these potential risk factors. This observation adds epidemiological evidence to the hypothesis that asthma and eczema may be related by other mechanisms than atopy.
